# Examining the Relationship Between Alcohol Use Disorder and Glaucoma: Protocol for a Scoping Review

**DOI:** 10.2196/76050

**Published:** 2025-12-31

**Authors:** Fatima Elghazali, Alexandria Hughes, Tracy Shields, Jennifer J Barb, Gwenyth R Wallen

**Affiliations:** 1 Translational Biobehavioral and Health Promotion Branch, Clinical Center, National Institutes of Health, 10 Center Dr, Bethesda, MD, 20892, United States, 1 301-435-9232; 2 NIH Research Library, National Institutes of Health, Bethesda, MD, United States

**Keywords:** alcohol use disorder, AUD, glaucoma, alcohol abuse, alcohol consumption

## Abstract

**Background:**

Alcohol use disorder (AUD) is a condition characterized by uncontrollable alcohol use despite negative social, occupational, or health consequences. AUD affects major organ systems in the body, including the eyes, in different ways. Alcohol seems to have dose-dependent effects on intraocular pressure (IOP), with some quantities lowering IOP and promoting blood flow to the optic nerve head, whereas higher quantities are linked to cardiovascular disorders and systemic physiological changes affecting glaucoma development. Current research shows mixed findings on the correlation between alcohol consumption and glaucoma, and little has been investigated in the AUD population. This paper outlines a protocol for a scoping review that aims to characterize the literature on the connection between AUD and its related conditions, such as alcohol dependence and alcohol misuse, and glaucoma and its associated symptoms, including increased IOP, optic nerve damage, and vision loss.

**Objective:**

The overarching goal of this scoping review is to synthesize an extensive overview of the current literature surrounding AUD or alcohol consumption and glaucoma. We aim to (1) map the existing literature on alcohol and glaucoma; (2) identify how alcohol consumption is associated with glaucoma; and (3) synthesize evidence concerning the association between AUD or alcohol consumption, including drinking frequency, quantity, and type, and glaucoma.

**Methods:**

A biomedical librarian will conduct a systematic search of PubMed and MEDLINE, Embase, Web of Science, and CENTRAL. A 2-step process will be used to screen the results, using Covidence as the screening software. All unique records retrieved from the databases and those identified through supplemental searches will be screened by 2 reviewers independently using the eligibility criteria. This will be followed by data charting. This evidence synthesis will summarize findings in narrative and tabular formats.

**Results:**

This scoping review was started in Covidence in November 2024 and is currently funded by the National Institutes of Health Intramural Research Program. The projected end date for data collection and submission is between November 2025 and January 2026.

**Conclusions:**

This scoping review aims to clarify the mixed findings on the association between AUD or alcohol consumption and glaucoma. The findings will guide future research in this area.

## Introduction

### Rationale

Alcohol use disorder (AUD) is a condition characterized by uncontrollable alcohol use despite negative social, occupational, or health consequences. The National Institute on Alcohol Abuse and Alcoholism defines heavy drinking as consuming 5 or more drinks on any day or 15 or more per week for men and consuming 4 or more drinks on any day or 8 or more drinks per week for women [1]. According to the 2023 National Survey on Drug Use and Health, 28.1 million adults ages 18 years and older had AUD in the previous year [2]. “AUD” is a newer term, as the disorder was previously called “alcoholism.” This change occurred in 2013 with the release of the *Diagnostic and Statistical Manual of Mental Disorders, Fifth Edition* [3]. Due to this terminology shift, we will include various terms for alcohol consumption in our study. Alcohol consumption is associated with risks of developing noncommunicable diseases such as liver and heart disease, cancers, and mental and behavioral conditions. The World Health Organization states that there is no form of alcohol consumption that is risk free [4]. The health effects of alcohol vary robustly based on the amount consumed. Excessive alcohol consumption can lead to alcoholic liver disease, cardiovascular disease, pancreatitis, cancers, psychiatric comorbidities, and more. Along with consumption levels, drinking patterns, such as light, moderate, and heavy drinking, strongly influence health outcomes [5].

The health consequences of alcohol are widely known as alcohol affects different major organs, including the eyes, based on a variety of factors, including age, sex, and other characteristics. For instance, chronic alcohol use has been reported to increase the risk of eye conditions such as age-related macular degeneration, diabetic retinopathy, cataract, optic neuropathy, retinal vascular and ocular surface diseases, and visual defects [6]. In a 2021 review of the literature, the risk of developing glaucoma from alcohol use was not clearly disclosed, but the review reinforced the need for investigation [6]. Glaucoma is an eye disease, as well as a group of eye diseases, that causes damage to the eye’s optic nerve due to fluid buildup in the front part of the eye. It is the leading cause of blindness for individuals aged >60 years [7]. Those with ocular hypertension or high intraocular pressure (IOP) are at a higher risk of glaucoma.

Glaucoma is the main cause of irreversible blindness worldwide. It is estimated that over 100 million individuals will have glaucoma in 2040 [8]. Alcohol is a potential modifiable risk factor for glaucoma, with some harmful associations reported at consumption levels below current US and UK alcohol consumption guidelines [9]. Glaucoma has long been known to be associated with alcohol consumption [10], as well as other risk factors such as IOP, age, gender, genetics and family history, race, and myopia [11]. Studies reporting the effects of alcohol on IOP vary in consistency [12]. Some suggest alcohol use is associated with an increased risk of elevated IOP depending on demographics and glaucoma status [13]. Current literature on the association between alcohol consumption or AUD and glaucoma, including the various subtypes of the disease, shows variable findings [6,14,15]. For instance, some papers report that daily alcohol consumption is a protective factor, associated with a decreased risk of glaucoma [16], whereas other studies report that greater total alcohol consumption is associated with a higher risk of exfoliation glaucoma and exfoliation glaucoma status risk [17]. Another review examined evidence from 10 studies that reported that habitual alcohol use is associated with higher IOP, a risk factor for glaucoma [15]. Most literature on the association between alcohol use and glaucoma is inconsistent.

In this review, we hope to summarize the associations between alcohol use and glaucoma categorically by glaucoma type and quantity of alcohol consumption as a comprehensive overview of the literature. A scoping review will be conducted, rather than a systematic review or meta-analysis, because the existing literature on AUD and glaucoma has not been characterized; other than a 2021 scoping review on drug use and ocular effects [18], there does not appear to be a scoping review specifically related to this topic. Taking a scoping review approach allows us to map the breadth of current evidence on the link between different levels of alcohol consumption or an AUD diagnosis and glaucoma, identify gaps in knowledge, and clarify key concepts in this area. Our aim is to provide a comprehensive overview that can inform the design of future systematic reviews or quantitative syntheses where the evidence base is more developed [19,20].

### Objectives

The primary research question and objectives of this review are to examine the existing literature on AUD, a relatively new term, and related alcohol use or consumption in relation to glaucoma risk. Our population of interest is individuals with AUD, alcohol misuse, alcohol abuse, and any terms that are synonymous with this condition. We plan to conduct a thorough review that also includes drinking patterns, demographics, and alcohol consumption levels.

## Methods

We will follow the methods outlined in the *JBI Manual for Evidence Synthesis* [21] and use the PRISMA-ScR (Preferred Reporting Items for Systematic Reviews and Meta-Analyses extension for Scoping Reviews) checklist [22] (Checklist 1) to report the completed review. This protocol was written using the PRISMA-ScR checklist as an outline, with additional information and details from Lely et al [23].

### Eligibility Criteria

The eligibility criteria were established by the study team to ensure inclusion of a broad yet relevant body of literature on the association between AUD and glaucoma. We sought to capture empirical research across diverse populations and study designs while excluding sources that would not directly inform the research question. A summary of inclusion and exclusion criteria is presented in Textbox 1.

Textbox 1. **Inclusion and exclusion criteria.**

**Inclusion criteria**

- Studies on individuals aged ≥18 years
- Patient populations of men and women
- Articles published in English
- Studies that document a diagnosis of glaucoma (*International Classification of Diseases, 10th Revision* code H40)
- Published articles including the year 2014 and up to 2025
- Primary research articles (case-control studies; case series or case reports; cohort studies; controlled trials; cross-sectional studies; prospective, longitudinal, and follow-up studies; and randomized controlled trials)

**Exclusion criteria**

- Animal or nonhuman studies
- Studies on individuals with other ocular conditions or diagnoses
- Diagnoses of congenital glaucoma (childhood glaucoma), including juvenile open-angle glaucoma, and any induced glaucoma (eg, drug, steroid, or corticosteroid induced)
- Cost-benefit analysis studies
- Commentaries or opinions, editorials, and letters
- Gray literature (conference proceedings, dissertations, ongoing clinical trials, and preprints)

### Search Strategy

The biomedical librarian (TS) will develop search strategies and incorporate team feedback on terminology. Four databases will be searched: PubMed, which includes MEDLINE (US National Library of Medicine); Embase (Elsevier); Web of Science Core Collection (Science Citation Index Expanded and Social Sciences Citation Index; Clarivate Analytics); and CENTRAL (Wiley). Search strategies include a combination of indexing terms (MeSH [Medical Subject Headings] and Emtree) and keywords (see Multimedia Appendix 1 for the complete search strategies). Concepts will be combined using the Boolean operators OR and AND, and the searches will be limited to English-language citations published since 2014. This date limit reflects diagnostic criteria as defined in the *Diagnostic and Statistical Manual of Mental Disorders, Fifth Edition* [3]. The searches will be peer reviewed by another biomedical librarian independent of this scoping review team following Peer Review of Electronic Search Strategies guidance [24], and the complete searches for every database will be documented as supplementary materials in the final manuscript to improve validity and reproducibility. All results will be exported to EndNote (version 21; Clarivate Analytics) and uploaded to the Covidence screening software (Veritas Health Innovation [25]), with duplicate citations removed.

### Study Selection

A 2-step process will be used to screen the results, using Covidence as the screening software. First, the titles and abstracts of all unique records retrieved from the databases and supplemental searches will be screened by 2 reviewers (FE and AH) independently using the eligibility criteria. A separate third reviewer (GRW) will resolve conflicts and, if necessary, discuss the conflict with at least one other reviewer to decide whether to include or exclude based on the eligibility criteria. Second, articles that meet the inclusion criteria after title and abstract screening will proceed to full-text screening. The PDFs of these articles will be obtained and uploaded into Covidence. Two reviewers (FE and AH) will independently screen the full texts using the same eligibility criteria. Conflicts will be resolved using the same process as before. Before working on the full review, reviewers will conduct a pilot of this 2-step process, including testing data extraction. For the pilot, the biomedical librarian (TS) will randomly select 50 articles from the completed searches and upload them to Covidence to test title and abstract screening and full-text screening steps with all reviewers (FE, AH, and GRW). On the basis of this pilot process, revisions or clarifications to the eligibility criteria, search strategies, and protocol will be made as needed.

### Data Charting

Covidence, as well as Microsoft Excel, will be used for data collection. Covidence will be used to screen titles and abstracts to filter out publications for the full-text review stage and then, finally, obtain a collection of papers for data extraction. Microsoft Excel will be used to collect the necessary information via an extraction template for the scoping review (Table 1). Two reviewers (FE and AH) will independently collect data from each included article. One reviewer (GRW) will check all collected data and reconcile any discrepancies in them. If data are missing, we will contact the corresponding author and, if no reply is received, mark the data as either missing or not reported. Similar to the screening pilot, a pilot of the data collection step will be completed with 3 reviewers (FE, AH, and GRW) on a sample of 2 to 5 records taken from the screening pilot. Changes to the data items or data collection process will be documented in the protocol.

**Table 1.** Data charting for the included literature.

Data item domain and subdomain	Description
Document characteristics	
Document type	Primary research articles
Title	Title of the publication
Authors	Authors of the publication
Publication year	Year of publication
Full citation	Citation of the publication
DOI	DOI included
Web link	Link to online source
Study characteristics	
Design	Case-control study, case report or series, or cohort study, among others
Setting	For example, hospital; inpatient or outpatient
Location	Country of publication
Population	Study eligibility criteria
Sample size	Number of participants in the study
Study objectives	Study research question (if relevant)
Characteristics of AUD**^a^**	
Drinking frequency	For example, number of drinks in a day, week, or month
Type of liquor consumed	For example, beer, wine, liquor, and other types of alcoholic beverages reported
Alcohol consumption level	For example, light, moderate, or heavy alcohol consumption
Alcohol consumption assessment tool	For example, self-reported surveys, questionnaires, and other assessment tools
Characteristics of glaucoma	
Glaucoma subtype	Types of glaucoma, such as open angle or normal tensions
IOP^b^ status or reading	For example, elevated, normal, or low (measured in mm Hg)
Co-use of substances	
Smoking (nicotine)	Smoking status: smoker, nonsmoker, or ex-smoker
Marijuana or cannabis	Marijuana or cannabis user, nonuser, or ex-user
Other outcomes	Any other study outcomes
Limitations	Limitations described by the authors and any other limitations identified
Implications and conclusions	Implications and conclusions as described by the authors
Vision characteristics	
Vision tests	IOP readings, OCT^c^, visual field and acuity, gonioscopy, and pachymetry

a AUD: alcohol use disorder.

b IOP: intraocular pressure.

c OCT: optical coherence tomography.

### Data Synthesis

As this is a scoping review, we will provide a narrative summary of our findings, including descriptive statistics on key data items. A PRISMA (Preferred Reporting Items for Systematic Reviews and Meta-Analyses) flow diagram will be used to document the disposition of results from the searches. We will include a table of key characteristics for the included studies and additional tables and figures for specific variables of interest as needed. We anticipate that studies may vary substantially in the details provided for alcohol consumption, such as providing intake in grams, drinks per day, or other unit of time or specific types of beverages, and we will describe consumption in the terms used in each study. We will synthesize findings while noting differences in exposure definitions and reference groups (eg, nondrinkers vs lowest intake category). Additionally, the studies each analyze cohorts and conclude whether alcohol intake is or is not associated with the glaucoma outcome; although the highest intake amount in each cohort will vary between studies, it will always be more relevant to each paper’s analyzed reference or control group (typically nondrinkers). We will synthesize findings noting this variability. Studies will also be categorized thematically and stratified into subgroups. Specifically, we will classify the included studies by (1) glaucoma subtype (eg, primary open-angle glaucoma, angle closure, or other specified types) and (2) drinking patterns (eg, light, moderate, or heavy) and alcohol consumption quantities as defined by each study.

This structured approach will allow us to identify recurring themes, highlight areas of evidence concentration versus gaps, and provide a comprehensive overview of the existing literature.

### Ethical Considerations

This review does not require ethics approval. We expect results from this scoping review to identify gaps in the current literature and encourage future research regarding patient advisement on the risks of alcohol consumption and the development of glaucoma. This review does not have any stakeholders. Results will be disseminated through peer-reviewed publications and conferences.

## Results

**Figure 1.**
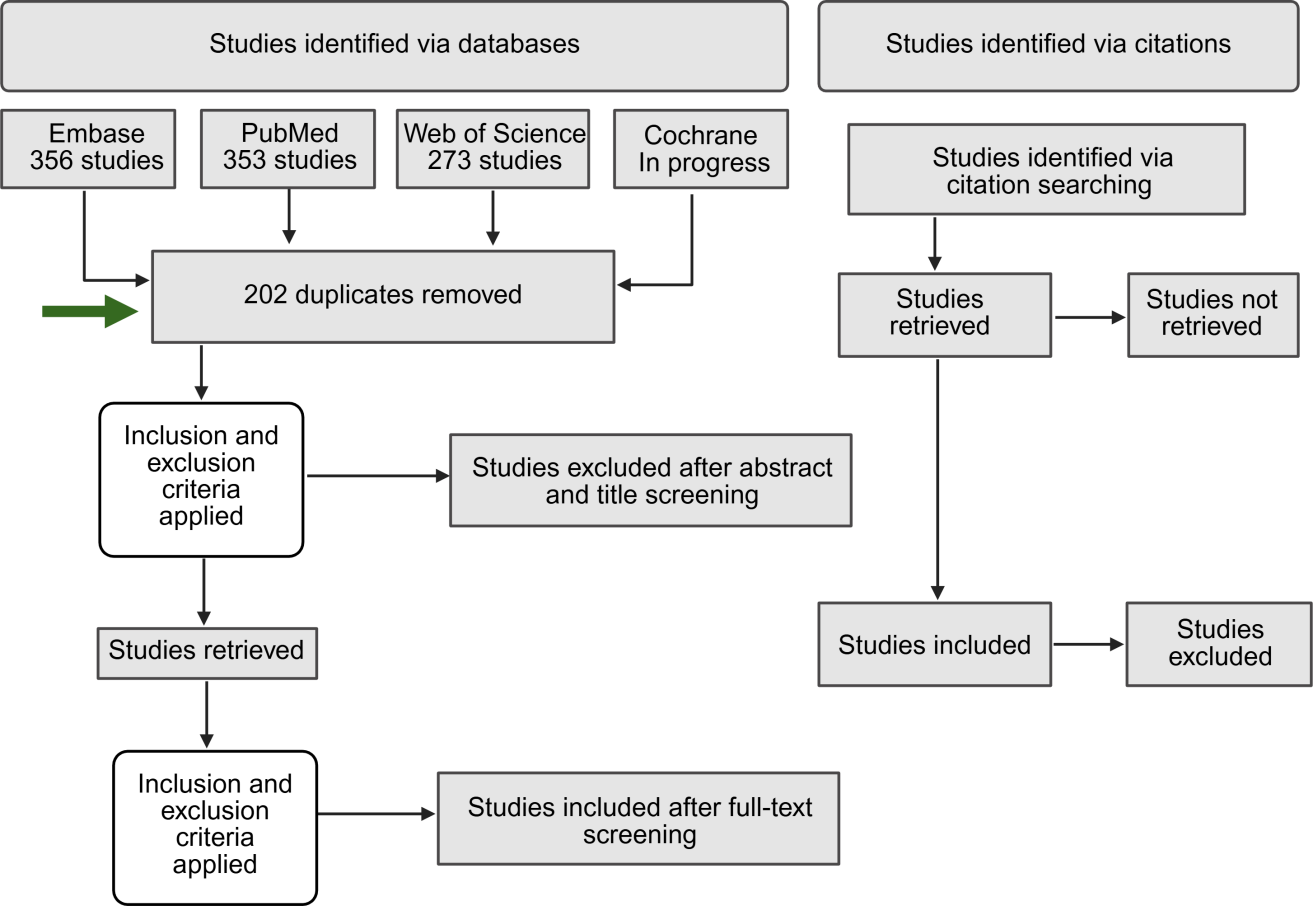
PRISMA-ScR (Preferred Reporting Items for Systematic Reviews and Meta-Analyses extension for Scoping Reviews) flowchart for the scoping review process.

This scoping review was started in Covidence in November 2024 and is currently funded by the National Institutes of Health’s Intramural Research Program. The database searches were completed and imported into Covidence and, in total, produced 982 papers, with 6 (0.6%) duplicates removed manually and 192 (19.6%) removed via Covidence for a total of 202 (20.6%) duplicates removed. Title and abstract screening of the remaining 780 articles is currently taking place. We will also be using the Cochrane database for our search, which may adjust these numbers and our screening process for the final manuscript. The projected end date for data collection and submission is between November 2025 and January 2026. The results will be presented in a final manuscript in relation to our research aims. We included a preliminary PRISMA-ScR flow diagram with our current database searches and a green arrow indicating our current project status (Figure 1). The final manuscript will include an updated PRISMA-ScR flow diagram, extraction table, and narrative description of our findings.

## Discussion

### Anticipated Findings

This scoping review is anticipated to reveal information linking alcohol consumption, including AUD diagnoses, to glaucoma. Evidence on this link has been heterogeneous at best. While some studies suggest that habitual alcohol intake is associated with elevated IOP or increased glaucoma risk, others have reported null or even protective associations. We expect that our mapping of the literature will highlight considerable variability in how alcohol exposure and glaucoma outcomes are defined and measured, which has likely contributed to the inconsistency of findings to date.

Our work will build on individual studies by providing a comprehensive overview of how alcohol use, from general consumption to clinically defined AUD, has been studied in relation to glaucoma and its subtypes. By consolidating and categorizing this evidence, the scoping review will clarify methodological patterns, illuminate conceptual gaps, and identify areas in which future systematic reviews or meta-analyses may be warranted. The aim of this work is to provide a comprehensive synthesis of the extensive literature on glaucoma and alcohol consumption, addressing the conflicting findings that have been reported. By thoroughly summarizing all relevant data, we intend to clarify the existing literature and address any gaps.

### Comparison to Prior Work

Previous studies have examined the relationship between alcohol and multiple other eye diseases with a brief overview of glaucoma but have been ultimately inconclusive or needed more information [6]. This review will add an extensive overview of the relationship between alcohol use and glaucoma, adding to existing findings by including details of alcohol consumption levels, drinking patterns, glaucoma types, and whether an association between alcohol use and glaucoma was statistically found. This offers a clear picture of the current knowledge on the association between alcohol use and glaucoma.

### Strengths and Limitations

A strength of this review is that it includes a comprehensive search method across multiple scientific databases for peer-reviewed literature to include in our review. Additionally, we will include literature that has been published in the last 10 years for the most up-to-date discoveries. Conducting a scoping review such as this one and following guidance from the PRISMA checklist and comprehensive search terms will increase transparency and reproducibility for future reviews. This approach offers an extensive overview of the most current knowledge on this topic.

We aim to be comprehensive in our scoping review; however, several limitations should be noted. First, we plan to synthesize data on glaucoma-related measures, such as IOP, optical coherence tomography, and visual field tests. These measures may not be consistently reported across the studies. It is also important to consider that alcohol consumption may be reported in varying ways between papers, which might limit comparability across studies. Second, restricting our review to English-language publications introduces the possibility of language bias as relevant studies published in other languages may be missed. Third, as with any review, there is potential for publication bias as studies with null or negative findings may be less likely to appear in the published literature. Fourth, even though our date limit of including literature from the past 10 years, from 2014 onward, may strengthen our study by including more up-to-date discoveries, it may also be a limitation due to missing important information or perspectives published prior to 2014. Finally, our exclusion of qualitative studies may introduce selection bias as insights from lived experience and patient perspectives will not be represented. Therefore, when evaluating the existing literature, we plan to take a holistic approach while also including any specific and relevant findings.

### Dissemination Plans

The dissemination methods for the findings of this scoping review will include publication in a peer-reviewed journal, which will start with manuscript submission planned for December 2026. Presentations at conferences both nationally and internationally are also planned, likely taking place before or around the time of manuscript submission.

### Conclusions

We intend to review the vast amount of literature related to the association between AUD and glaucoma to provide a summary of current knowledge and an evidence-based resource that may benefit physicians, patient care teams, and other health professionals in advising, treatment, and preventative care. While synthesizing the breadth of evidence, we will concurrently identify gaps in the literature, thus identifying areas that require future focused research efforts.

## Supplementary material

10.2196/76050 Multimedia Appendix 1 Search strategies.

10.2196/76050 Checklist 1 PRISMA-ScR checklist.

## Abbreviations

AUD: alcohol use disorder
IOP: intraocular pressure
MeSH: Medical Subject Headings
PRISMA: Preferred Reporting Items for Systematic Reviews and Meta-Analyses
PRISMA-ScR: Preferred Reporting Items for Systematic Reviews and Meta-Analyses extension for Scoping Reviews
