# Supplementary material for: Examining the Relationship Between Alcohol Use Disorder and Glaucoma: Protocol for a Scoping Review
Source: JMIR Res Protoc. 2025 Dec 31;14:e76050. doi: 10.2196/76050 (PMC12809109; doi:10.2196/76050)
Supplement: Multimedia Appendix 1 [file resprot-v14-e76050-s001.docx]

**Supplemental Material**

**Search strategy for PubMed, which includes MEDLINE**
**Provider/Platform:** US National Library of Medicine (NLM); <https://pubmed.ncbi.nlm.nih.gov/>  
**Applied Settings and Limits:** PubMed Advanced Search Builder; no other limits

| Set | Concept | Search Strategy |
| --- | --- | --- |
| #1 | alcohol use disorder | ("Alcohol-Related Disorders"[Mesh:NoExp] OR "Alcoholism"[Mesh] OR "Alcoholic Intoxication"[Mesh] OR "Binge Drinking"[Mesh] OR "Alcohol Drinking"[Mesh] OR "alcohol use disorder*"[tiab] OR "alcohol* relat*"[tiab] OR "alcohol* induc*"[tiab] OR "alcohol* spectr*"[tiab] OR "alcohol* drink*"[tiab] OR "alcoholic*"[tiab] OR "alcoholism*"[tiab] OR "alcohol* intoxicat*"[tiab] OR "alcohol* use"[tiab] OR "alcohol* misus*"[tiab] OR "alcohol* abus*"[tiab] OR "alcohol* dependen*"[tiab] OR "alcohol* addict*"[tiab] OR "addict* alcohol*"[tiab] OR "bing* drink*"[tiab] OR "drink* bing*"[tiab] OR "bing* alcohol*"[tiab] OR "alcohol* bing*"[tiab] OR "excess* alcohol*"[tiab] OR "excess* drink*"[tiab] OR "alcohol* consum*"[tiab]) |
| #2 | glaucoma | ("Glaucoma"[Mesh] OR "glaucoma*"[tiab] OR "Intraocular Pressure"[Mesh] OR "intraocular*"[tiab] OR "intra-ocular*"[tiab] OR "intraorbital*"[tiab] OR "intra-orbital*"[tiab] OR "eye pressure*"[tiab] OR "eyeball pressure*"[tiab] OR "eye ball pressure*"[tiab] OR "ocular pressure*"[tiab] OR "ocular tension"[tiab] OR "optic nerve*"[tiab] OR "second cranial nerve"[tiab] OR "cranial nerve II"[tiab] OR "cranial nerve 2"[tiab:~0] OR "cranial nerve two"[tiab:~0]) |
| #3 | alcohol use disorder AND glaucoma | #1 AND #2 |

**Search strategy for Embase**
**Provider/Platform:** Elsevier; <https://www.embase.com/>  
**Applied Settings and Limits:** Advanced Search; limit to Embase and exclude MEDLINE (these limits remove most duplicates with PubMed)

| Set | Concept | Search Strategy |
| --- | --- | --- |
| #1 | alcohol use disorder | ('alcoholism'/exp OR 'alcohol abuse'/exp OR 'alcohol intoxication'/exp OR 'alcohol consumption'/exp OR 'alcohol use disorder*':ti,ab,kw OR 'alcohol* relat*':ti,ab,kw OR 'alcohol* induc*':ti,ab,kw OR 'alcohol* spectr*':ti,ab,kw OR 'alcohol* drink*':ti,ab,kw OR 'alcoholic*':ti,ab,kw OR 'alcoholism*':ti,ab,kw OR 'alcohol* intoxicat*':ti,ab,kw OR 'alcohol* use':ti,ab,kw OR 'alcohol* misus*':ti,ab,kw OR 'alcohol* abus*':ti,ab,kw OR 'alcohol* dependen*':ti,ab,kw OR 'alcohol* addict*':ti,ab,kw OR 'addict* alcohol*':ti,ab,kw OR 'bing* drink*':ti,ab,kw OR 'drink* bing*':ti,ab,kw OR 'bing* alcohol*':ti,ab,kw OR 'alcohol* bing*':ti,ab,kw OR 'excess* alcohol*':ti,ab,kw OR 'excess* drink*':ti,ab,kw OR 'alcohol* consum*':ti,ab,kw) |
| #2 | glaucoma | ('glaucoma'/exp OR 'glaucoma*':ti,ab,kw OR 'intraocular pressure'/exp OR 'intraocular*':ti,ab,kw OR 'intra-ocular*':ti,ab,kw OR 'intraorbital*':ti,ab,kw OR 'intra-orbital*':ti,ab,kw OR 'eye pressure*':ti,ab,kw OR 'eyeball pressure*':ti,ab,kw OR 'eye ball pressure*':ti,ab,kw OR 'ocular pressure*':ti,ab,kw OR 'ocular tension':ti,ab,kw OR 'optic nerve*':ti,ab,kw OR 'second cranial nerve':ti,ab,kw OR 'cranial nerve ii':ti,ab,kw OR 'cranial nerve 2':ti,ab,kw OR 'cranial nerve two':ti,ab,kw) |
| #3 | alcohol use disorder AND glaucoma | #1 AND #2 |
| #4 | #3 limited to Embase records and exclude MEDLINE records | #3 AND ([embase]/lim NOT [medline]/lim) |

**Search strategy for Web of Science Core Collection: Science Citation Index Expanded** (1900 to present); Social Sciences Citation Index (1900 to present) 
**Provider/Platform:** Clarivate Analytics; <https://www.webofscience.com/wos/woscc/advanced-search>  
**Applied Settings and Limits:** Advanced Search, exact phrase toggled on; no other limits

| Set | Concept | Search Strategy |
| --- | --- | --- |
| #1 | alcohol use disorder | TS=("alcohol use disorder*" OR "alcohol* relat*" OR "alcohol* induc*" OR "alcohol* spectr*" OR "alcohol* drink*" OR "alcoholic*" OR "alcoholism*" OR "alcohol* intoxicat*" OR "alcohol* use" OR "alcohol* misus*" OR "alcohol* abus*" OR "alcohol* dependen*" OR "alcohol* addict*" OR "addict* alcohol*" OR "bing* drink*" OR "drink* bing*" OR "bing* alcohol*" OR "alcohol* bing*" OR "excess* alcohol*" OR "excess* drink*" OR "alcohol* consum*") |
| #2 | glaucoma | TS=("glaucoma*" OR "intraocular*" OR "intra-ocular*" OR "intraorbital*" OR "intra-orbital*" OR "eye pressure*" OR "eyeball pressure*" OR "eye ball pressure*" OR "ocular pressure*" OR "ocular tension" OR "optic nerve*" OR "second cranial nerve" OR "cranial nerve II" OR "cranial nerve 2" OR "cranial nerve two") |
| #3 | alcohol use disorder AND glaucoma | #1 AND #2 |

**Search strategy for Cochrane Central Register of Controlled Trials (CENTRAL)**
**Provider/Platform:** Wiley/Cochrane Library; <https://www.cochranelibrary.com/central>
**Applied Settings and Limits:** Search Manager; no other limits

| Set | Concept | Search Strategy |
| --- | --- | --- |
| #1 | alcohol use disorder | ([mh ^"Alcohol-Related Disorders"] OR [mh Alcoholism] OR [mh "Alcoholic Intoxication"] OR [mh "Binge Drinking"] OR [mh "Alcohol Drinking"] OR ("alcohol use" NEXT disorder*):ti,ab OR (alcohol* NEXT relat*):ti,ab OR (alcohol* NEXT induc*):ti,ab OR (alcohol* NEXT spectr*):ti,ab OR (alcohol* NEXT drink*):ti,ab OR alcoholic*:ti,ab OR alcoholism*:ti,ab OR (alcohol* NEXT intoxicat*):ti,ab OR (alcohol* NEXT "use"):ti,ab OR (alcohol* NEXT misus*):ti,ab OR (alcohol* NEXT abus*):ti,ab OR (alcohol* NEXT dependen*):ti,ab OR (alcohol* NEXT addict*):ti,ab OR (addict* NEXT alcohol*):ti,ab OR (bing* NEXT drink*):ti,ab OR (drink* NEXT bing*):ti,ab OR (bing* NEXT alcohol*):ti,ab OR (alcohol* NEXT bing*):ti,ab OR (excess* NEXT alcohol*):ti,ab OR (excess* NEXT drink*):ti,ab OR (alcohol* NEXT consum*):ti,ab) |
| #2 | glaucoma | ([mh Glaucoma] OR glaucoma*:ti,ab OR [mh "Intraocular Pressure"] OR intraocular*:ti,ab OR intra-ocular*:ti,ab OR intraorbital*:ti,ab OR intra-orbital*:ti,ab OR ("eye" NEXT pressure*):ti,ab OR ("eyeball" NEXT pressure*):ti,ab OR ("eye ball" NEXT pressure*):ti,ab OR ("ocular" NEXT pressure*):ti,ab OR "ocular tension":ti,ab OR ("optic" NEXT nerve*):ti,ab) |
| #3 | alcohol use disorder AND glaucoma | #1 AND #2 |
